# Sex differences in machine learning computed tomography-derived fractional flow reserve

**DOI:** 10.1038/s41598-022-17875-9

**Published:** 2022-08-16

**Authors:** Mahmoud Al Rifai, Ahmed Ibrahim Ahmed, Yushui Han, Jean Michel Saad, Talal Alnabelsi, Faisal Nabi, Su Min Chang, Myra Cocker, Chris Schwemmer, Juan C. Ramirez-Giraldo, William A. Zoghbi, John J. Mahmarian, Mouaz H. Al-Mallah

**Affiliations:** 1 Houston Methodist Debakey Heart & Vascular Center, 6550 Fannin Street, Houston, TX 77030 USA; 2 University of Kentucky, Lexington, KY USA; 3 Computed Tomography-Research Collaborations, Siemens Healthineers, Malvern, PA USA; 4 Computed Tomography-Research & Development, Siemens Healthcare GmbH, Forchheim, Germany

**Keywords:** Cardiovascular diseases, Ischaemia

## Abstract

Coronary computed tomography angiography (CCTA) derived machine learning fractional flow reserve (ML-FFR_CT_) can assess the hemodynamic significance of coronary artery stenoses. We aimed to assess sex differences in the association of ML-FFR_CT_ and incident cardiovascular outcomes. We studied a retrospective cohort of consecutive patients who underwent clinically indicated CCTA and single photon emission computed tomography (SPECT). Obstructive stenosis was defined as ≥ 70% stenosis severity in non-left main vessels or ≥ 50% in the left main coronary. ML-FFR_CT_ was computed using a machine learning algorithm with significant stenosis defined as ML-FFR_CT_ < 0.8. The primary outcome was a composite of death or non-fatal myocardial infarction (D/MI). Our study population consisted of 471 patients with mean (SD) age 65 (13) years, 53% men, and multiple comorbidities (78% hypertension, 66% diabetes, 81% dyslipidemia). Compared to men, women were less likely to have obstructive stenosis by CCTA (9% vs. 18%; p = 0.006), less multivessel CAD (4% vs. 6%; p = 0.25), lower prevalence of ML-FFR_CT_ < 0.8 (39% vs. 44%; p = 0.23) and higher median (IQR) ML-FFR_CT_ (0.76 (0.53–0.86) vs. 0.71 (0.47–0.84); p = 0.047). In multivariable adjusted models, there was no significant association between ML-FFR_CT_ < 0.8 and D/MI [Hazard Ratio 0.82, 95% confidence interval (0.30, 2.20); p = 0.25 for interaction with sex.]. In a high-risk cohort of symptomatic patients who underwent CCTA and SPECT testing, ML-FFR_CT_ was higher in women than men. There was no significant association between ML-FFR_CT_ and incident mortality or MI and no evidence that the prognostic value of ML-FFR_CT_ differs by sex.

## Introduction

Atherosclerotic cardiovascular disease (ASCVD) remains the leading cause of morbidity and mortality among women^1^. Women typically present later in life and have atypical symptoms. Furthermore, women are less likely to be referred for diagnostic procedures and receive guideline-directed medical treatment and lifestyle interventions^2–5^. Despite having less obstructive coronary artery disease (CAD), women often have worse outcomes than men^6^. Non-invasive assessment of CAD in women may be limited by smaller body habitus, reduced peak exercise achievement, and breast attenuation.

Coronary computed tomography angiography (CCTA) offers the advantage of direct visualization of the coronary tree without the inherent risks of invasive angiography and with acceptable accuracy^7^. However, similar to invasive coronary angiography (ICA), CCTA does not provide information of the hemodynamic significance of coronary stenoses. Recent advances in computational fluid dynamics have enabled the non-invasive assessment of coronary blood flow using CCTA derived fractional flow reserve (FFR_CT_). FFR_CT_ has been shown to improve the diagnostic accuracy of ischemia compared to other noninvasive tests^8^. FFR_CT_ has also been suggested to play a role as both a gatekeeper to ICA by decreasing the likelihood of nonobstructive CAD at ICA^9^, and as guide to revascularization as it has been shown to predict standard of care guided coronary revascularization^10^. Importantly, FFR_CT_ has similar discriminatory power for detection of ischemia by ICA regardless of sex^11^.

Machine learning based FFR_CT_ (ML-FFR_CT_) is the latest tool to non-invasively determine coronary blood flow without the need for high computation demand or off-site data transfer. Although not approved for clinical use, a recent meta-analysis has shown how sensitivity and specificity are comparable to both computational flow and gold standard invasive angiography-based determination of FFR^12^.

A prior study from the Assessing Diagnostic Value of Non-invasive FFRCT in Coronary Care (ADVANCE) registry found that women have higher FFR_CT_ compared to men for the same degree of coronary stenosis^13^. Among patients with positive FFR_CT_ (< 0.80) women were found to have less obstructive CAD and less revascularization. However, the study did not assess the association of FFR_CT_ with incident cardiovascular outcomes. In the present analysis, we compare machine learning FFR_CT_ among men and women to determine whether there are sex differences in the distribution of ML-FFR_CT_ by degree of coronary stenosis and specific coronary vessel involvement. We also evaluate sex differences in the prognostic significance of ML-FFR_CT_ in relation to incident cardiovascular outcomes.

## Methods

### Study population

Our study population consisted of 471 consecutive patients who underwent a clinically indicated CCTA and single positron emission computed tomography (SPECT) for suspected CAD between January 1, 2016 to June 22, 2020 at a large referral center.

This cohort of patients was initially established to assess the comparative prognostic role of functional assessment with SPECT vs ML-FFR_CT_ when added to CCTA anatomic assessment, which we have published before^14^. Dual SPECT-CCTA testing was done at the discretion of the treating physician. In the current sub-analysis, we present sex-specific differences. A flow diagram is provided (Supplementary Fig. 1).

Approval from the Institutional Review Board at the Houston Methodist Academic Institute was obtained prior to the start of the study. Informed consent was waived by Institutional Review Board at the Houston Methodist Academic Institute due to the retrospective nature of the study. All methods were carried out in accordance with relevant guidelines and regulations.

### Assessment of covariates

Information on sociodemographic variables, medical history, comorbidities and medication use was obtained using chart review of electronic health records within 30 days of imaging.

### Follow-up and outcome

The primary outcome was death or non-fatal myocardial infarction (D/MI). Secondary outcome included major adverse cardiovascular events (MACE), a composite of death, MI, and unplanned revascularization—PCI or CABG occurring more than 90 days after index imaging). Myocardial infarction was defined as the 4th universal definition of Myocardial Infarction^15^. All outcomes were obtained from chart review and adjudicated by expert physicians in a blinded manner. All patients were followed from the date of CCTA imaging to either the occurrence of outcomes or the last known date of contact noted on the patient records.

### CCTA

CCTA scans were performed using 3rd generation SOMATOM FORCE Scanner (Siemens, Forchheim, Germany). Image acquisition was performed in accordance with the Society of Cardiovascular Computed Tomography (SCCT) guidelines and has been described before^14,16^. Briefly, intravenous metoprolol was administered for patients with a heart rate ≥ 65 beats/min and sublingual nitroglycerin 0.4 mg was administered immediately before image acquisition. During image acquisition, 60–100 cc of contrast was injected, followed by saline flush. Axial scans were obtained with prospective electrocardiographic gating. Image acquisition was performed to include the coronary arteries, left ventricle and proximal ascending aorta.

Images were analyzed with a 3-dimensional workstation using one of several post-processing methods including axial, multiplanar reformat, maximum intensity projection and cross-sectional analysis. Type and location of lesion were visually evaluated using an 18-segment model according to SCCT guidelines^16^. In each segment, atherosclerosis was defined as tissue structures > 1 mm^2^ within the coronary artery lumen or adjacent to the lumen that could be discriminated from pericardial tissue, epicardial fat or vessel lumen itself. Coronary stenosis was classified as none (0%), mild (1–49%), moderate (50–69%), or severe (≥ 70%) based on degree of narrowing of the luminal diameter. Anatomically obstructive CAD by CCTA was defined at 70% stenosis severity in non-left main vessels and 50% in the left main artery. Findings were reported using SCCT Coronary Artery Disease Reporting & Data System (CAD-RADS). All interpretations were done by experienced imaging cardiologists (at least 10 years of experience).

### ML-FFR_CT_

ML-FFR_CT_ was determined using a machine learning based computation of fractional flow reserve (ML-FFR_CT_, cFFR 3.2, Siemens Healthcare GmbH, Forchheim, Germany, not available for commercial use). Methods for ML-FFR_CT_ determination have been described before^14^. The coronary tree was isolated semi-automatically to generate a 3-dimensional coronary model. All vessels and branches with a diameter of at least 2 mm were included. ML-FFR_CT_ was determined at the midpoint of a segment for normal segments and 1 cm distal to stenosis for segments with lesions based on prior work showing higher prognostic role of measurements distal to stenosis^17^. Details on the derivation of ML-FFR_CT_ values, diagnostic accuracy and concordance of results with invasive ML-FFR_CT_ have been reported previously^12,18^. A quantitative 3-dimensional model of the coronary tree was reconstructed and a 17-segment model was used. Image processing was done by two investigators blinded to results from other tests. Both investigators underwent extensive training prior to image processing. The reproducibility of findings was assessed on a random subset of patients and was found to be high (ICC 0.981 per patient and 0.970 per segment, absolute mean difference 0.019 per and 0.027 per segment) and across image quality and degrees of stenosis by CAD-RADS. The minimum ML-FFR_CT_ values were recorded in each segment per patient and summarized using median (interquartile range) and categorized. Hemodynamically significant ischemia was defined as ML-FFR_CT_ < 0.8 consistent with prior literature^19–21^.

### Statistical analysis

Continuous variables were presented as mean (standard deviation)/median (inter-quartile range) and categorical variables were presented as count (percentage) stratified by sex. Results were compared using Student’s t test for continuous normally distributed variables and median testing for continuous non-normally distributed variables or chi-square test for categorical variables.

Median (IQR) and per-patient minimum ML-FFR_CT_ were calculated for each CAD-RAD category (0, 1–2, 3, 4A and 4B) per patient and for each coronary vessel and stratified by sex to unmask potential differences by severity of stenosis. The prevalence of ML-FFR_CT_ < 0.8 was similarly calculated for each CAD-RAD category and coronary vessel stratified by sex. The results were graphically depicted using bar charts and box plots as appropriate.

Multivariable-adjusted Cox proportional hazards models were used to study the association between ML-FFR_CT_ < 0.80 and incident outcomes stratified by sex after confirming the proportionality assumption using log–log plots. Models were adjusted for age, hypertension, diabetes mellitus, dyslipidemia, ever cigarette smoking, indication for CCTA testing, early revascularization (PCI or CABG within 90 days of testing), and degree of coronary stenosis by CCTA. Results were further stratified by CAD-RAD score (≤ 2 vs. > 2). In sensitivity analyses, we utilized median per-patient ML-FFR_CT_ and lower thresholds (significant ML-FFR_CT_ defined as < 0.75 and < 0.70).

To determine whether there should be separate cutoffs for ML-FFR_CT_ for men versus women, mean ML-FFR_CT_ for each coronary vessel proximal, mid, and distal segment were summarized for the overall study cohort and stratified by sex. Difference across segments was assessed using the Kruskal–Wallis equality of proportions rank test.

All analyses were conducted using Stata 17.0 (StataCorp, College Station, Texas) and a p-value of < 0.05 was considered statistically significant.

## Results

The study population consisted of 471 patients with mean (SD) age 64 (13) years, 47% women, and multiple comorbidities (78% hypertension, 66% diabetes, 81% dyslipidemia). The median number of days between SPECT and CCTA was 24 days (IQR 3–118 days) and nearly two-thirds of the cohort had CCTA prior to or on the same day as SPECT (337 CT then SPECT vs 134 SPECT then CT). Women had a similar distribution of cardiovascular risk factors except they were less likely to report history of smoking (19% vs. 31%). Compared to men, women were less likely to have obstructive stenosis (9% vs. 18%; p = 0.006), less often had advanced (higher CAD-RADS) stenosis and less likely to have multivessel CAD (4% vs. 6%; p = 0.25) (Tables 1, 2).

**Table 1:** Baseline characteristics of the study population by sex.

Sociodemographic	Total	Sex		
		Male	Female	p
	N = 471	N = 249	N = 222	
Age, N (%)	63.83 (12.60)	63.30 (12.70)	64.42 (12.48)	0.34
**Comorbidities**				
Hypertension, N (%)	369 (78.3%)	199 (79.9%)	170 (76.6%)	0.38
Diabetes, N (%)	309 (65.6%)	160 (64.3%)	149 (67.1%)	0.51
Dyslipidemia, N (%)	382 (81.1%)	202 (81.1%)	180 (81.1%)	0.99
Ever smoker, N (%)	118 (25.1%)	77 (30.9%)	41 (18.5%)	0.002
**Symptoms**				
Chest pain or shortness of breath, N (%)	279 (59.2%)	140 (56.2%)	139 (62.6%)	0.16
**Medication**				
Aspirin/clopidogrel, N (%)	372 (79.0%)	198 (79.5%)	174 (78.4%)	0.76
Statin, N (%)	349 (74.1%)	184 (73.9%)	165 (74.3%)	0.92
ACE/ARB, N (%)	295 (62.6%)	152 (61.0%)	143 (64.4%)	0.45
Beta blockers, N (%)	357 (75.8%)	190 (76.3%)	167 (75.2%)	0.78
Calcium channel blockers, N (%)	199 (42.3%)	105 (42.2%)	94 (42.3%)	0.97

p-value comparing women and men was calculated using Student’s t test for continuous normally-distributed variables and median testing for continuous non-normally distributed variables or chi-square test for categorical variables.

*ACE* Angiotensin converting enzyme, *ARB* angiotensin receptor blocker.

**Table 2:** Imaging characteristics of the study population by sex.

	Total	Sex		
		Male	Female	p
**CCTA stenosis**				
CCTA CAD-RAD, N (%)				< 0.001
CAD-RAD 0	115 (24.4%)	44 (17.7%)	71 (32.0%)	
CAD-RAD 1/2	208 (44.2%)	108 (43.4%)	100 (45.0%)	
CAD-RAD 3	76 (16.1%)	49 (19.7%)	27 (12.2%)	
CAD-RAD 4A	50 (10.6%)	33 (13.3%)	17 (7.7%)	
CAD-RAD 4B	22 (4.7%)	15 (6.0%)	7 (3.2%)	
CCTA obstructive stenosis, N (%)	64 (13.6%)	44 (17.7%)	20 (9.0%)	0.006
CCTA multi-vessel disease, N (%)	25 (5.3%)	16 (6.4%)	9 (4.1%)	0.25
**FFRct**				
**Minimum FFRct**				
Minimum FFRct per patient, median (IQR)	0.74 (0.51–0.85)	0.71 (0.47–0.84)	0.76 (0.53–0.86)	0.047
**Ischemic stenosis**				
ML-FFRct < 0.70 on any proximal/mid segment, N (%)	120 (25.5%)	67 (26.9%)	53 (23.9%)	0.45
ML-FFRct < 0.75 on any proximal/mid segment, N (%)	147 (31.2%)	85 (34.1%)	62 (27.9%)	0.15
ML-FFRct < 0.80 on any proximal/mid segment, N (%)	196 (41.6%)	110 (44.2%)	86 (38.7%)	0.23

p-value comparing women and men was calculated using Student’s t test for continuous normally-distributed variables and median testing for continuous non-normally distributed variables or chi-square test for categorical variables.

*CCTA* Coronary computed tomography angiography, *FFR*_*CT*_ fractional flow reserve derived using computed tomography.

**Figure 1:**
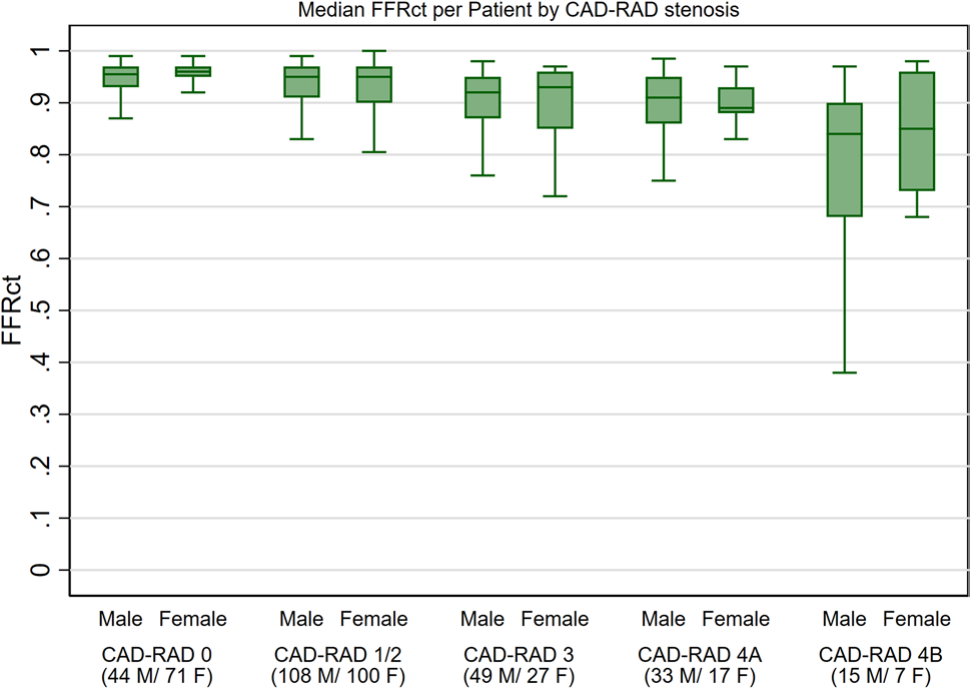
Median FFR_CT_ per patient and for each coronary vessel stratified by sex and CAD-RAD score. *FFR*_*CT*_ Fractional flow reserve derived using computed tomography, *M* male, *F* female.

**Figure 2:**
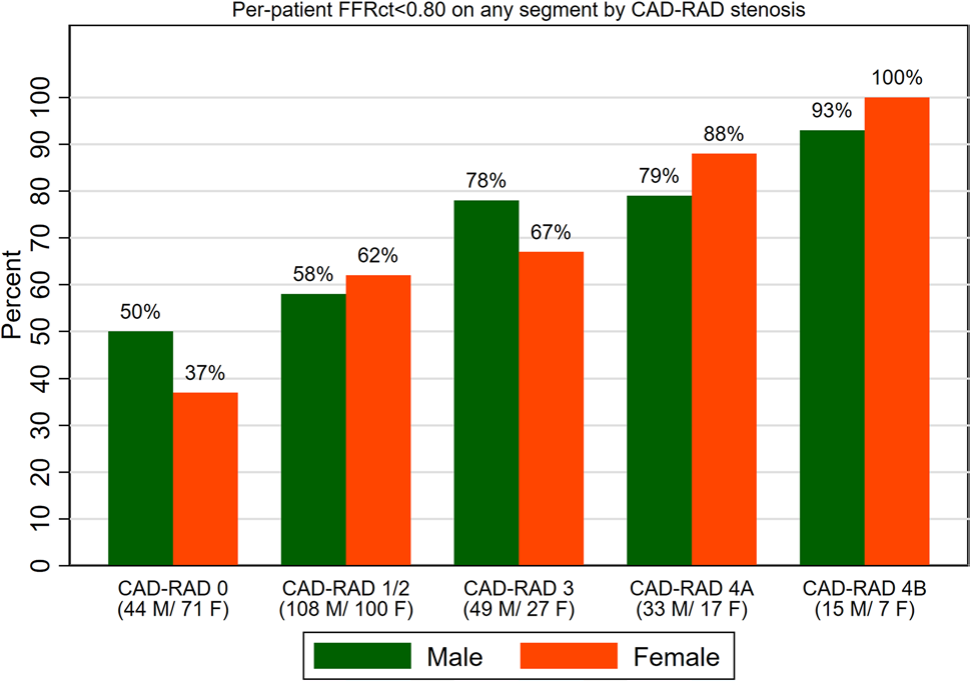
Prevalence of FFR_CT_ < 0.8 in any segment per patient and for each coronary vessel stratified by sex and CAD-RAD score. *FFR*_*CT*_ Fractional flow reserve derived using computed tomography, *LAD* left anterior descending artery, *LCX* left circumflex artery, *RCA* right coronary artery, *M* male, *F* female.

Women had higher median (IQR) ML-FFR_CT_ compared to men: 0.76 (0.53–0.86) vs. 0.71 (0.47–0.84; p = 0.047). In stratified results, median ML-FFR_CT_ was generally higher among women compared to men regardless of CAD-RADS score or specific vessel involvement (Fig. 1; Supplementary Fig. 2). Results were similar comparing per-patient minimum FFR_CT_ stratified by CAD-RADS (Supplementary Fig. 3). The prevalence of ML-FFR_CT_ < 0.8 was lower among women compared to men (39% vs. 44% respectively; p = 0.23). No major differences were seen when stratifying by CAD-RADS. (Fig. 2; Supplementary Fig. 4). Sensitivity analysis using lower cut-offs showed similar results (Supplementary Fig. 5). Similarly, no major differences were seen in per-patient minimum FFR_CT_ values when stratified by CAD-RADS (Supplementary Table 1). While non-significant, FFR_CT_ was lower among women with significant coronary stenosis (CAD-RAD 4A/4B).

Over a median follow-up time of 18 months, there were 33 incident D/MI (38 D/20 MI) and 38 MACE (38 D/20 MI/42 PCI/5 CABG) events which corresponded to 3.48 and 4.05 events per 1000 person-years respectively (Table 3). There was no significant difference in event rates between men and women. In multivariable adjusted models, there was no significant association between ML-FFR_CT_ < 0.8 and D/MI: Hazard Ratio 0.82, 95% confidence interval (0.30, 2.20); p = 0.254 for interaction with sex (Table 4). There was no significant association in analyses stratified by CAD-RADS score (≤ 2 vs. > 2). Similar results were observed for the secondary outcome of MACE (Table 4) and when significant ML-FFR_CT_ was defined as < 0.70 or < 0.75 (results not shown).

**Table 3:** Outcomes of the study population by sex.

	Total	Sex		
		Male	Female	p
**Outcomes**				
Death/MI				
Incidence Rate (per 1000 person-year)	2.89	3.55	2.25	0.204
N (%)	33 (7.0%)	20 (8.0%)	13 (5.9%)	0.36
**MACE**				
Incidence rate (per 1000 person-year)	3.36	4.32	2.43	0.086
N (%)	38 (8.1%)	24 (9.6%)	14 (6.3%)	0.18
All-cause death, N (%)	20 (4.2%)	12 (4.8%)	8 (3.6%)	0.51
Myocardial infarction, N (%)	13 (2.8%)	8 (3.2%)	5 (2.3%)	0.53
PCI 90-days post imaging, N (%)	42 (8.9%)	22 (8.8%)	20 (9.0%)	0.95
CABG 90-days post imaging, N (%)	5 (1.1%)	4 (1.6%)	1 (0.5%)	0.22

p-value comparing women and men was calculated using Student’s t test for continuous normally-distributed variables and median testing for continuous non-normally distributed variables or chi-square test for categorical variables.

*MACE* major adverse cardiovascular events, *MI* myocardial infarction, *PCI* percutaneous coronary intervention, *CABG* coronary artery bypass graft.

**Table 4:** Hazard ratios for the association of ML-FFR_CT_ < 0.8 and incident outcomes.

	Death or all-cause mortality			Major adverse cardiovascular outcomes		
	Unadjusted	Adjusted	p for interaction	Unadjusted	Adjusted	p for interaction
Overall	1.87	0.82	0.690	1.69	0.95	0.921
CAD-RAD ≤ 2	2.44	1.22	0.781	2.44	1.23	0.770
CAD-RAD > 2	0.82	0.30	0.112	0.67	0.51	0.283

ML-FFR_CT_ refers to fractional flow reserve derived using computed tomography and was categorized as < 0.8

Models were adjusted for age, hypertension, diabetes mellitus, dyslipidemia, ever cigarette smoking, indication for CCTA testing, early revascularization (PCI or CABG within 90 days of testing), and degree of coronary stenosis by CCTA.

p-for interaction refers to multiplicative interaction between ML-FFR_CT_ and sex (women vs. men).

**Figure 3:**
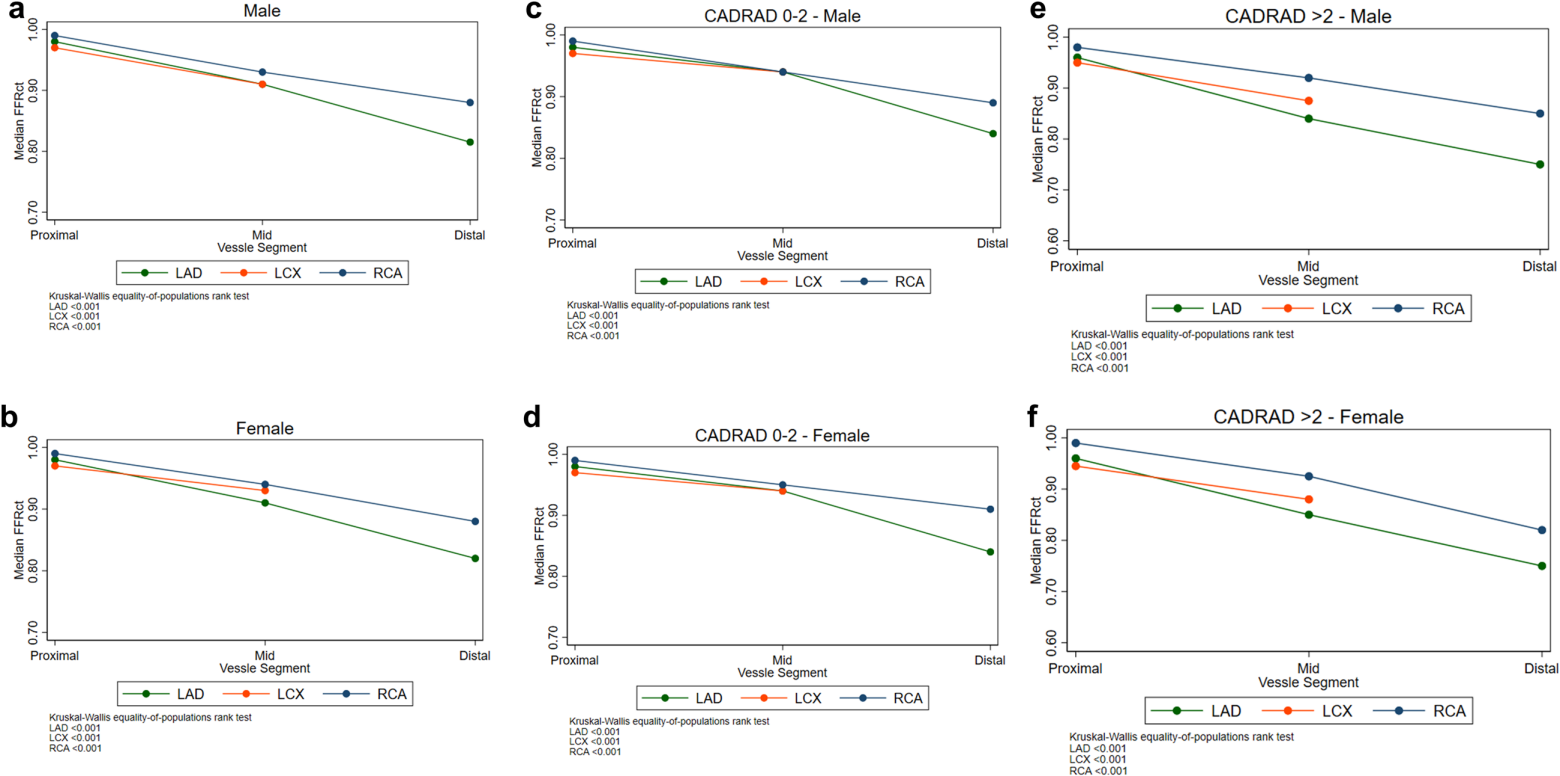
Proximal-to-distal median FFR_CT_ stratified for each coronary vessel stratified by sex (**a**,**b**) and CAD-RAD score (**c**–**f**). *FFR*_*CT*_ Fractional flow reserve derived using computed tomography, *LAD* left anterior descending artery, *LCX* left circumflex artery, *RCA* right coronary artery, *M* male, *F* female.

Median ML-FFR_CT_ decreased moving from proximal to distal segments in each coronary vessel for both men and women (Fig. 3). These results were similar stratifying by CAD-RADS score (≤ 2 vs. > 2). In multivariable adjusted models, there was no significant association between median ML-FFR_CT_ with the primary and secondary outcome (Supplementary Table 2).

## Discussion

In our study of high-risk stable CAD patients who underwent concomitant CCTA and SPECT testing, we found that women had higher ML-FFR_CT_ when compared to men but this was likely due to a lower burden of obstructive coronary atherosclerotic disease. However, hemodynamically significant stenosis as defined by ML-FFR_CT_ < 0.8 was higher among women than men for advanced degrees of stenosis (i.e. CAD-RAD 4A-4B). ML-FFR_CT_ was not associated with incident adverse outcomes and there was no significant interaction with sex.

Women constituted half of our study population and had a similar cardiovascular risk factor profile compared to men except for lower prevalence of smoking. Despite having higher ML-FFR_CT_, less obstructive CAD, and less advanced stenosis in our study, women experienced similar rates of death and mortality compared to men in our study. While the prevalence of ML-FFR_CT_ < 0.8 was higher among women, analyses stratified by degree of coronary stenosis revealed that women more likely to have hemodynamically significant coronary stenosis among those with more advanced stenosis burden (CAD-RAD 4A/B), which might have contributed to excess event rates in this group. This discordance points to the importance of coronary CT perfusion data that is afforded by ML-FFR_CT_. Lastly, we did not find that the prognostic value of ML-FFR_CT_ differs by sex and there does not appear to be a sex-specific cut-off for ML-FFR_CT_. Taken together, our results suggest that ML-FFR_CT_ has similar prognostic value among women and men.

Women are less likely to achieve maximum exercise capacity on exercise stress testing and more likely to have common attenuation artifacts that can reduce the diagnostic performance of SPECT for diagnosing CAD^22^. Therefore, a complementary anatomical (CCTA) and functional approach (FFR_CT_) may improve the sensitivity of coronary CT for diagnosing obstructive CAD among women. Improving the diagnostic accuracy of CAD among women can help decrease the excess burden of CAD among women and poorer outcomes compared to men^6^.

Few randomized controlled studies have evaluated the use of CCTA vs. standard of care (including ICA and SPECT) in patients with stable chest pain. The SCOT-HEART trial found that CCTA did improve clinical outcomes^23^, whereas use of CCTA in the PROMISE trial did not^24^. In the latter study there was no evidence of a significant interaction of CCTA with sex suggesting a similar prognostic value of CCTA in both men and women. A functional evaluation of coronary stenosis using FFR_CT_ may help further improve diagnostic accuracy of CCTA. In the FAME 2 trial of patients with stable chest pain, invasive FFR-guided PCI decreased the rate of urgent revascularization among those with functionally significant stenosis. These results were similar among men and women without significant interaction by sex^25^.

Knegt et al. found that CT perfusion resulted in improved discrimination compared with CCTA alone for the diagnosis of hemodynamically significant CAD defined by FFR and quantitative ICA^26^. In the CREDENCE trial, CCTA was superior to SPECT for predicting invasive FFR though addition of FFR_CT_ to atherosclerotic plaque predictors did not significantly improve discrimination^27^. The AUC for CCTA was similar for men and women (0.83 vs. 0.88) but sex-stratified results for FFR_CT_ were not presented in that study. In the ReASSESS Study, FFR_CT_ and SPECT were similar in identifying hemodynamically significant stenosis^21^. However, FFR_CT_ was more sensitive than SPECT in the overall study population and in both men and women suggesting similar prognostic value by sex.

In a subgroup analysis from the ADVANCE registry, FFR_CT_ was higher among women compared to men regardless of the degree of coronary stenosis suggesting that women have less hemodynamically significant lesions^13^. Similarly we found that ML-FFR_CT_ was higher among women though functionally significant FFR_CT_ was higher among women with a higher burden of stenosis. This was a surprise finding as while women tend to have less obstructive CAD compared to men, a higher degree of stenosis may be more hemodynamically significant in women as our study indicates. This could be related to the very high prevalence of comorbidities in our study population (for example 66% vs. 22% diabetes) and subsequent higher event rates compared to the ADVANCE registry (4% death, 3% MI vs. event rates of 0.6–1.16%)^26,27^ However, there was no significant association between FFR_CT_ and incident outcomes in our study either in the overall population or among those with higher degree of coronary stenosis.

An interesting finding is the high rates of hemodynamically significant stenosis in those with non-obstructive stenosis (CAD-RADS 0–2). Our results are similar to those from prior studies using the only FFR_CT_ tool approved for clinical use where ischemia was detected in 33% of vessels with normal CCTA and 42% with mild (1–49%) stenosis^28^.

### Limitations

Our study is not without limitations. Our patients likely constitute a particularly high-risk population as they were referred to concomitant CCTA and SPECT testing and therefore results may not be generalizable to otherwise lower risk patients. We used a machine learning prototype to measure FFR_CT_ which is not currently validated or widely adopted in clinical practice. We did not evaluate for plaque characteristics such as plaque volume which could have affected analysis of FFR_CT_ by sex. Our study was likely underpowered to evaluate incident outcomes given the small number of events our patients experienced as well as the relatively short duration of follow-up. There was no information on cause of death so we could not determine the association of FFR_CT_ with cardiac-specific mortality outcomes. Similarly, we could not identify if the culprit vessel had the lowest ML-FFR_CT_ in patients with an MI because such data was not available for our cohort. Patients with non-obstructive stenosis (CAD-RADS 0–2) were included primarily for comparability with prior studies that also included patients with similar range of stenosis^28,29^. ML-FFR_CT_ could not be processed in 12% of the cohort. This rate is similar to those from prior studies using computational flow-based determination^9,30,31^. We used chart review to determine baseline characteristics and outcomes, potentially resulting in under-detection. However, obituaries and linked electronic records from other institutions were checked for patients who had not been seen at our institution for more than a year. Lastly, we cannot exclude the possibility of residual confounding.

## Conclusion

In conclusion, among a high-risk cohort of symptomatic patients who underwent CCTA and SPECT testing, the degree of coronary atherosclerotic disease was lower and ML-FFR_CT_ was higher in women than men. There is no significant association between FFR_CT_ and incident mortality or MI and no evidence that the prognostic value of ML-FFR_CT_ differs by sex.

## Supplementary Information

Supplementary Information.

## Data Availability

Deidentified data for this study will be made available by the corresponding author upon reasonable request.
