# Supplementary material for: Sex differences in machine learning computed tomography-derived fractional flow reserve
Source: Sci Rep. 2022 Aug 16;12:13861. doi: 10.1038/s41598-022-17875-9 (PMC9381799; doi:10.1038/s41598-022-17875-9)
Supplement: Supplementary file 1 — Supplementary Information. [file 41598_2022_17875_MOESM1_ESM.docx]

**Supplemental Figure Legend**


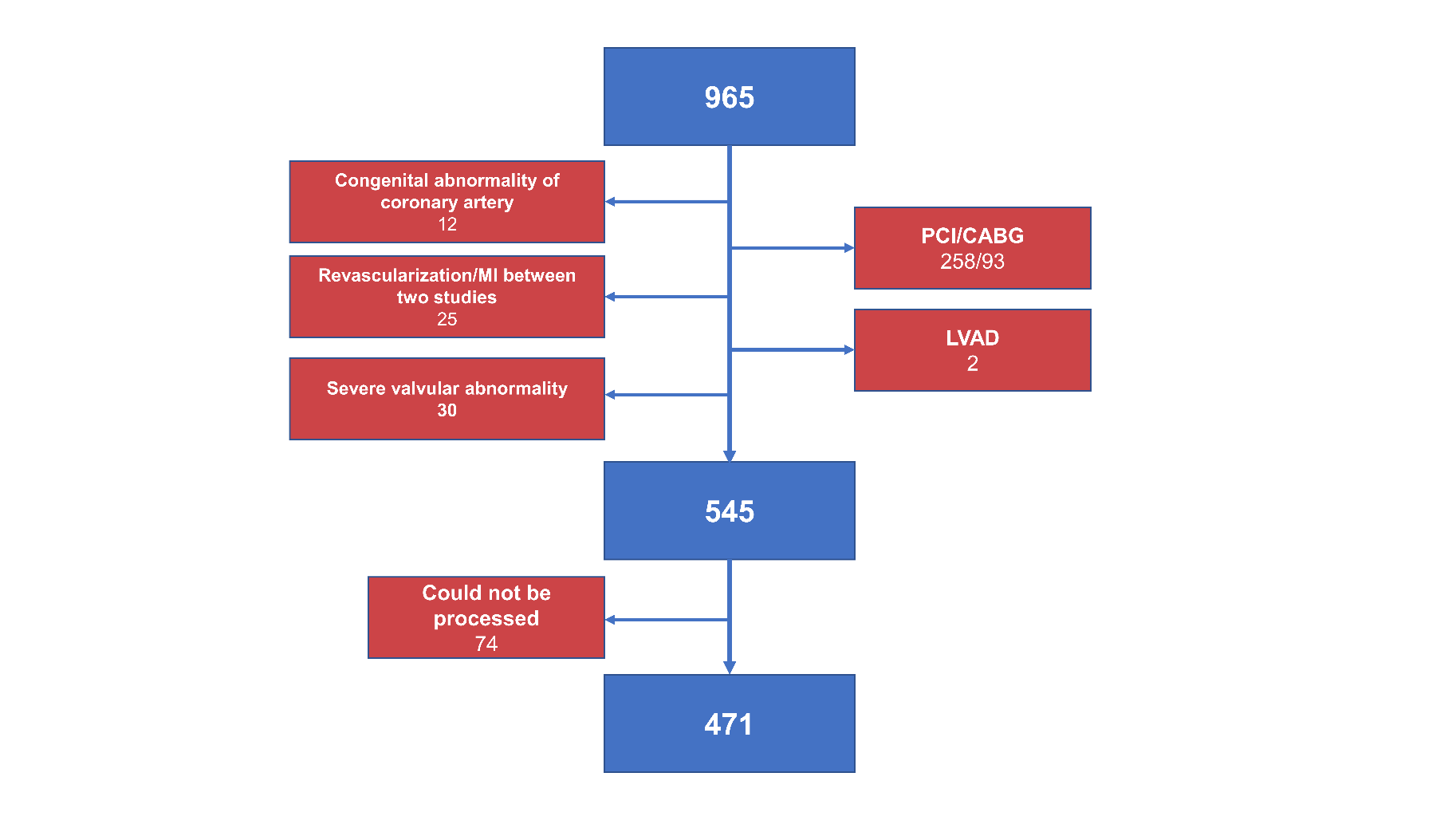


***Supplemental Figure 1*** Flow diagram

Abbreviations: CABG – coronary artery bypass graft; LVAD – left ventricular assist device; MI – myocardial infarction; PCI – percutaneous coronary intervention


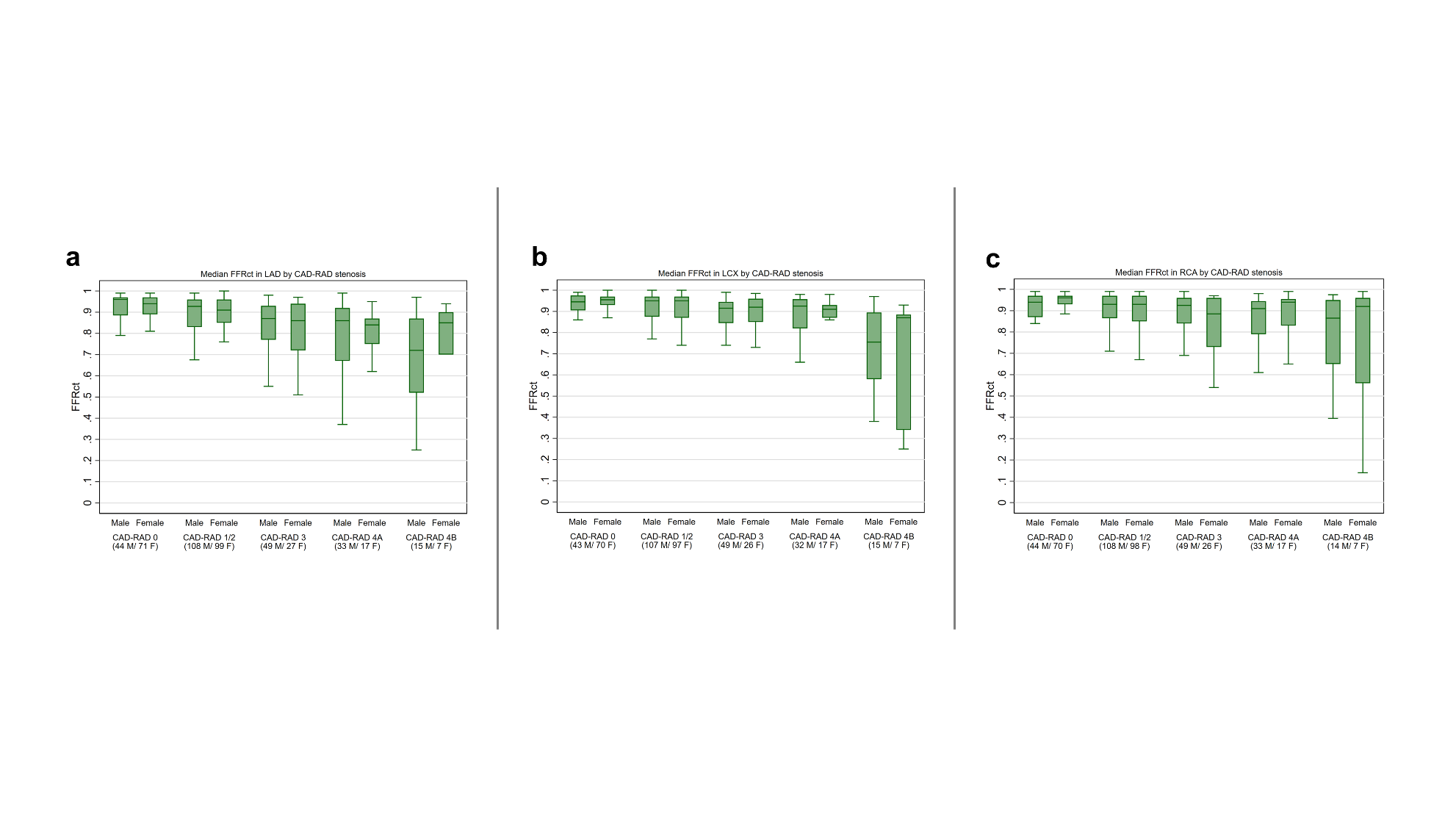


***Supplemental Figure 2*** Median FFR_CT_ for each coronary vessel stratified by sex and CAD-RAD score

Abbreviations: CAD-RAD: Coronary Artery Disease Reporting & Data System; FFR_CT_: Fractional Flow Reserve derived using Computed Tomography; LAD: Left Anterior Descending artery; LCX: Left Circumflex Artery; RCA: Right Coronary Artery


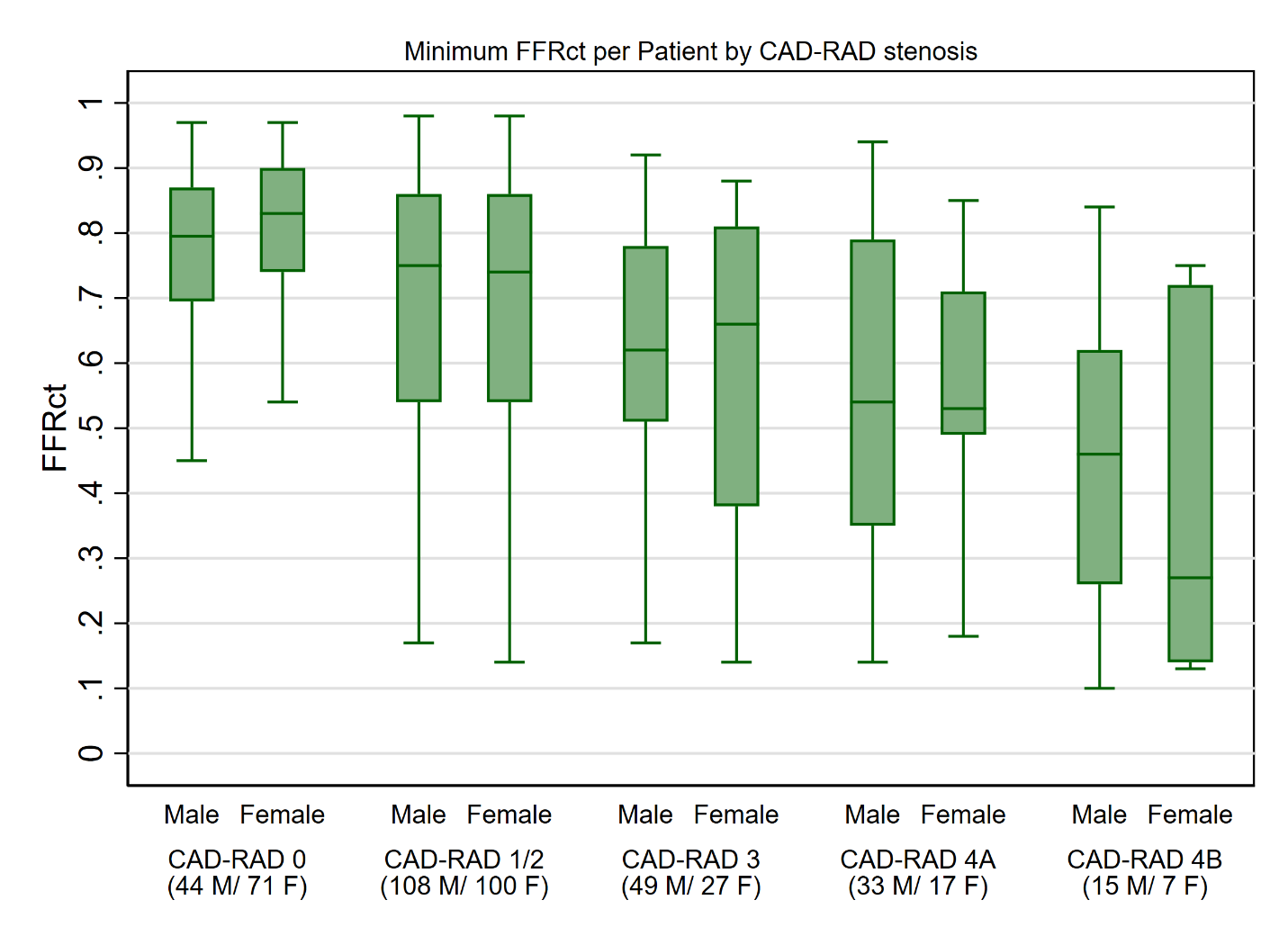


***Supplemental Figure 3*** Minimum FFR_CT_ per patient and for each coronary vessel stratified by sex and CAD-RAD score

Abbreviations: CAD-RAD: Coronary Artery Disease Reporting & Data System; FFR_CT_: Fractional Flow Reserve derived using Computed Tomography;


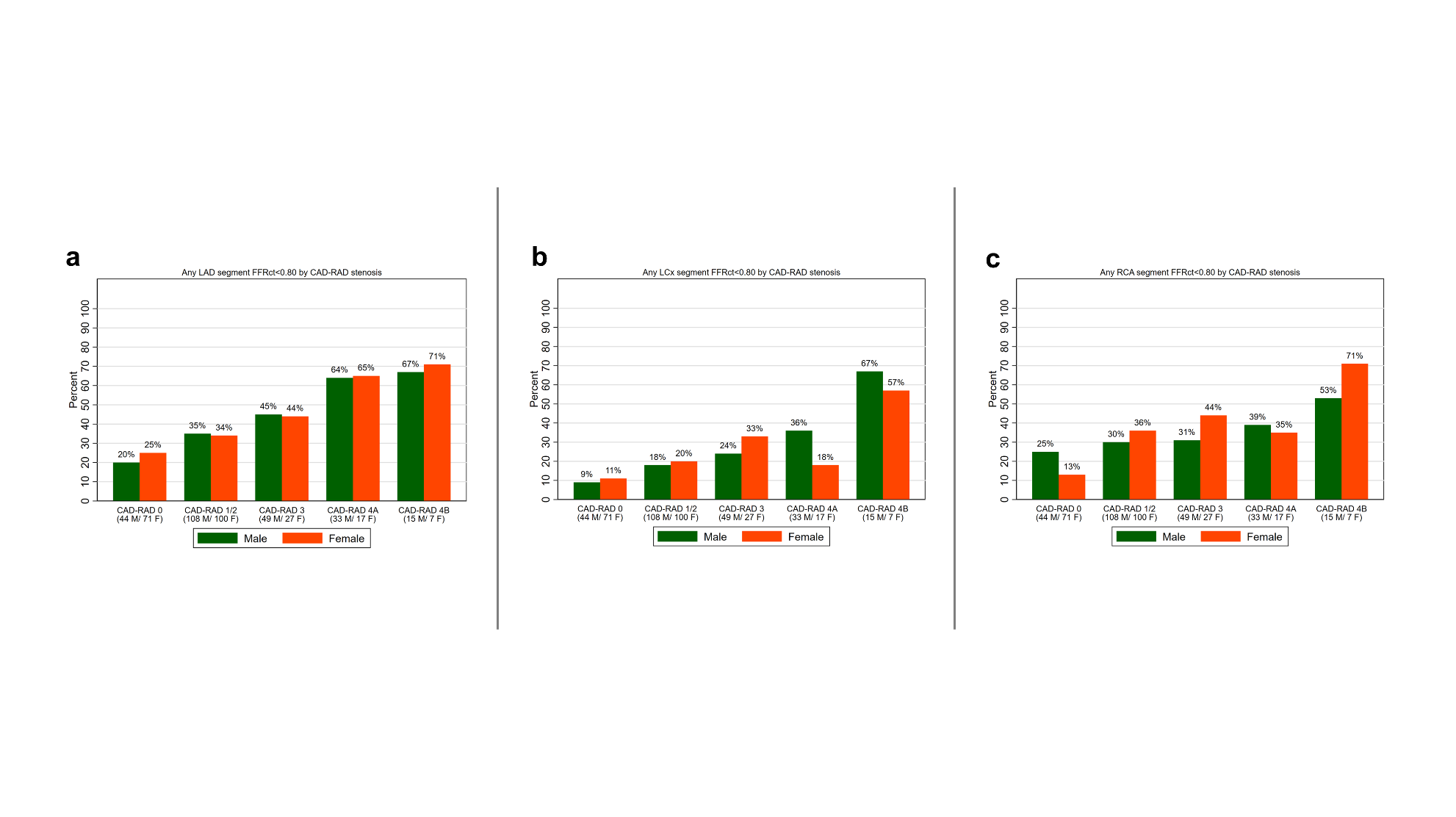


***Supplemental Figure 4*** Prevalence of FFR_CT_ <0.8 in any segment for each coronary vessel stratified by sex and CAD-RAD score

Abbreviations: FFR_CT_: CAD-RAD: Coronary Artery Disease Reporting & Data System; Fractional Flow Reserve derived using Computed Tomography; LAD: Left Anterior Descending artery; LCX: Left Circumflex Artery; RCA: Right Coronary Artery; M: Male; F: Female


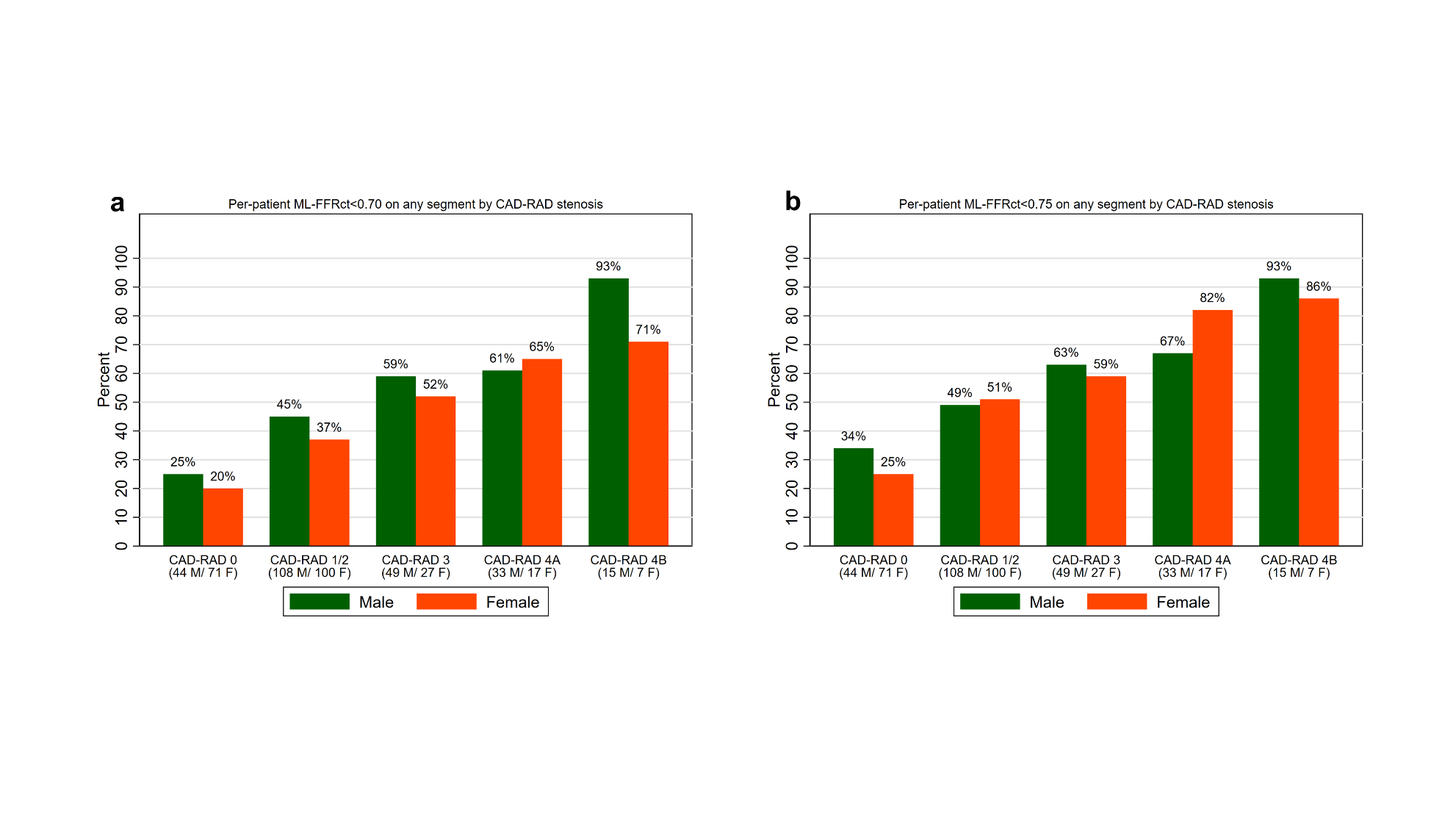


***Supplemental Figure 5*** Prevalence of FFR_CT_ <0.7 (3a) and <0.75 (3b) in any segment in any segment per patient stratified by sex and CAD-RAD score

Abbreviations: CAD-RAD: Coronary Artery Disease Reporting & Data System FFR_CT_: Fractional Flow Reserve derived using Computed Tomography; LAD: Left Anterior Descending artery; LCX: Left Circumflex Artery; RCA: Right Coronary Artery; M: Male; F: Female

***Supplemental Table 1*** Per-patient minimum FFR_CT_ values stratified by sex and CAD-RAD score

|  |  | **Sex** | | |
| --- | --- | --- | --- | --- |
|  |  |  | | |
|  | **Total** | ***Male*** | ***Female*** | ***P*** |
| **Per-patient minimum FFR_CT_** |  |  |  |  |
| CCTA CAD-RAD |  |  |  |  |
| CAD-RAD 0 | 0.82 (0.72 - 0.9) | 0.79 (0.69 - 0.87) | 0.83 (0.74 - 0.9) | 0.034 |
| CAD-RAD 1/2 | 0.74 (0.54 - 0.86) | 0.75 (0.54 - 0.86) | 0.74 (0.54 - 0.86) | 0.89 |
| CAD-RAD 3 | 0.64 (0.47 - 0.8) | 0.62 (0.51 - 0.78) | 0.66 (0.38 - 0.81) | 0.632 |
| CAD-RAD 4A | 0.53 (0.36 - 0.77) | 0.54 (0.35 - 0.79) | 0.53 (0.49 - 0.71) | 0.559 |
| CAD-RAD 4B | 0.42 (0.24 - 0.62) | 0.46 (0.26 - 0.62) | 0.27 (0.14 - 0.72) | 0.99 |

Abbreviations: CAD-RAD: Coronary Artery Disease Reporting & Data System; Coronary CCTA: Coronary Computed Tomography Angiography; FFRCT: Fractional Flow Reserve derived using Computed Tomography;

| ***Supplemental Table 2.*** Hazard ratios for the association of median ML-FFR_CT_ and incident outcomes | | | | | | |
| --- | --- | --- | --- | --- | --- | --- |
|  | **Death or all-cause mortality** | | | **Major adverse cardiovascular outcomes** | | |
|  | **Unadjusted** | **Adjusted** | **P for interaction** | **Unadjusted** | **Adjusted** | **P for interaction** |
| **Overall** | 0.03 | 0.30 | 0.910 | 0.03 | 0.21 | 0.953 |
| **CAD-RAD ≤2** | 0.02 | 9.64 | 0.195 | 0.02 | 9.30 | 0.196 |
| **CAD-RAD >2** | 0.15 | 0.25 | 0.521 | 0.24 | 0.17 | 0.134 |
| ML-FFR_CT_ refers to the median Fractional Flow Reserve derived using Computed Tomography | | | | | | |
| Models were adjusted for age, hypertension, diabetes mellitus, dyslipidemia, ever cigarette smoking, indication for CCTA testing, early revascularization (PCI or CABG within 90 days of testing), and degree of coronary stenosis by CCTA. | | | | | | |
| P-for interaction refers to multiplicative interaction between ML-FFR_CT_ and sex (women vs. men) | | | | | | |
